# Effects of Evidence-Based Fall Reduction Programing on the Functional Wellness of Older Adults in a Senior Living Community: A Clinical Case Study

**DOI:** 10.3389/fpubh.2016.00262

**Published:** 2016-12-22

**Authors:** Andrew Harnish, William Dieter, Albert Crawford, Tiffany E. Shubert

**Affiliations:** ^1^Fox Rehabilitation, Cherry Hill, NJ, USA; ^2^Thomas Jefferson University, Philadelphia, PA, USA; ^3^Shubert Consulting, Chapel Hill, NC, USA

**Keywords:** group-based exercise, falls, stratification, evidence-based, wellness

## Abstract

**Background:**

Older adults at a high risk of falls may be referred to a physical therapist. A physical therapy episode of care is designed for the transition of an older adult from a high fall risk to a moderate to low fall risk. However, these episodes of care are limited in time and duration. There is compelling evidence for the efficacy of group-based exercise classes to address risk, and transitioning an older adult from physical therapy to a group-based program may be an effective way to manage risk through the continuum of care.

**Objectives:**

The purpose of this study was to translate research findings into a “real world” setting, and demonstrate the efficacy of integrating evidence-based fall prevention exercises into pre-existing exercise classes at a senior living facility as a “proof of concept” model for future programing.

**Methods:**

Twenty-four participants aged 65 years and older living in a senior living community and the community were stratified into group-based exercise classes. Cutoff scores from functional outcome measures were used to stratify participants. Exercises from The Otago Exercise Program were implemented into the classes. Functional outcome measures collected included the 10-Meter Walk Test, 30-Second Sit to Stand, and Timed Up and Go (TUG). Number of falls, hospitalizations, and physical therapy episodes of care were also tracked. Data were compared to a control group in a different senior living community that offered classes with similar exercises aimed at improving strength and mobility. The classes were taught by an exercise physiologist and were of equal duration and frequency.

**Results:**

Participants demonstrated significant improvements in all functional outcome measures. TUG mean improved from 13.5 to 10.4 s (*p* = 0.034). The 30-Second Sit to Stand mean improved from 10.5 to 13.4 (*p* = 0.002). The 10-Meter Walk Test improved from 0.81 to 0.98 m/s (*p* < 0.0001). Participants did not experience any falls or hospitalizations, and two participants required physical therapy episodes of care.

**Conclusion:**

Implementing an evidence-based fall reduction program into a senior living program has a positive effect on strength, balance, fall risk, gait speed, fall rate, hospitalizations, and amount of physical therapy intervention.

## Introduction

According to the Centers for Disease Control and Prevention (CDC), falls are the leading cause of injury among adults aged 65 years and older in the United States (1). Each year, more than one out of four older adults will fall in the United States, with the total number of falls in the millions (2). Furthermore, 20–30% suffer moderate to severe injuries that will greatly impact their functional mobility and independence (3).

Fall injuries are among the 20 most expensive medical conditions in the United States. In 2013, the total direct medical costs of falls were $34 billion (4). By 2020, the direct and indirect cost of fall injuries is projected to reach $67.7 billion (5). Medicare currently pays for about 77% of the costs of falls (4). Private insurance (12%), self-pay (3%), Medicaid (2%), and other sources account for the rest (4). Medicare costs in the first year after a fall average between $13,797 and $20,450 (5). By 2030, Medicare is expected to reach solvency (6). Therefore, it is imperative that physical therapists and other health-care professionals are proactive to implement programs aimed at decreasing falls and controlling their costs.

Managing and treating the growing older adult population is both complex and challenging. By the year 2030, the expected number of adults aged 65 or older in the United States is expected to nearly double to 72.1 million (7). As the health-care field evolves, it is now more important than ever for physical therapists to provide client-centered care of the highest quality and value to maximize outcomes and reduce costs. Physical therapists play a central role in screening for fall risk, diagnosing balance and/or gait impairments, and providing treatment strategies that provide optimal dosage and intensity.

Unfortunately, many insurance companies in the United States do not reimburse for many evidence-based fall prevention programs recommended as best practice to maximally reduce falls and fall risk (8). As a result, the physical therapy profession must be driven to innovate and to affect change in ways that will allow us to provide valuable services that are not only evidence-based but cost effective to both payers and most importantly, the consumer. As physical therapists, many of the “consumers” are part of the baby boomer generation. This population continues to grow, and many older adults are living longer with the presence of multiple comorbidities.

In 2015, Stubbs et al. published an umbrella review of meta-analyses of randomized controlled trials that investigated any intervention to prevent falls in community-dwelling older adults aged 60 or older (9). The authors concluded that exercise, as well as multifactorial interventions prevented falls, including the risk, odds, and rate of falls. The authors defined exercise as “physical therapy based exercises” and “exercises focused on gait, balance, and functional mobility” (9). This review coincides with the landmark Cochrane review performed by Gillespie et al. which concluded that group and home-based exercise programs reduce the rate of falling and the risk of falling (10).

Physical therapists play an integral role on the multidisciplinary team focused on reducing falls and hospitalizations. Clients are typically prescribed a series of exercises to improve their strength, mobility, and balance. Unfortunately, adherence to home exercise programs (HEPs) is typically low and gains from therapy are not maintained once the client is discharged (11). Fortunately, group-based exercise classes, as demonstrated by Stubbs et al., have been shown to maintain benefits gained from therapy, and to have positive effects on fall rate, functional mobility, balance, health-related quality of life, and fear of falling (9).

Although physical therapists possess the clinical knowledge and skill to design group-based exercise classes, these classes are typically not offered by physical therapists for a variety of reasons, such as limited time, resources, and lack of reimbursement. However, such classes can be made feasible with the assistance of qualified health-care extenders to conduct classes, such as exercise physiologists. These types of programs show promise to facilitate the transition after a physical therapy episode of care and to continue to improve clinical outcomes. For a group program to be most effective, it must integrate evidence-based components.

In 2008, Sherrington et al. conducted a systematic review of 44 studies covering 9603 participants (12). Exercise programs had an overall 17% reduction in fall rates compared to control non-exercise groups (12). However, when used together, three factors proved to be most efficacious in reducing falls by up to 42%. They were as follows: (1) exercise must provide a moderate or high challenge to balance and must include a combination of reducing the base of support, movement of the center of mass, and reducing upper extremity support (12); (2) exercise must be of a sufficient dose to have an effect, specifically, total dose more than 50 h, equating to 2 h per week for 6 months (12); and (3) absence of a walking program specifically as an intervention. The authors hypothesized that this was due to time taken away from high challenge balance training (12).

In 2011, Sherrington et al. released best practice recommendations to guide the use of exercise for falls prevention. In addition to their original findings, Sherrington et al. includes that ongoing exercise is necessary or benefits are lost once exercise is terminated and that these exercises may be undertaken in a group or home-based setting (13). Group-based exercise classes that are offered year round and maximize Sherrington’s three factors may provide a feasible way to reduce falls to more at-risk individuals.

The high dosage of 2 h per week and supervision required for safe and effective interventions may pose large financial burdens and administrative barriers for payors and facilities (14). However, recent research has shown that group-based exercise can decrease direct medical costs for individuals, while also providing a better allocation of economic resources and achieve the same or better outcomes (15, 16). In the United States’ current health-care reimbursement model (fee for service), this may prove to be a feasible way to provide fall prevention exercise on a larger and more cost-effective scale. Attending a group-based exercise class in conjunction with therapy services allows participants to achieve and maintain the dosage recommendations proposed by Sherrington to maximally reduce falls. A well-designed class allows its participants to maintain an optimal level of function, which in turn may help reduce the recidivism often seen in geriatric physical therapy. If participants are able to stay healthier and reduce falls, injuries, and hospitalizations, this can prove to be a large saving to the health-care system. In fact, a study by Hektoen et al. which followed women older than 80 years old concluded that the health-care costs per individual for treating a fall-related injury were 1.85 times greater than the cost of implementing a fall prevention program (17).

Martin et al. conducted a systematic review of the effectiveness of physical therapist-administered group-based exercise on fall prevention in ambulatory adults greater than 65 years old living in the community or in an institution (14). The authors reported that compared to a non-exercise group, the exercise group demonstrated significant improvements in the following outcomes: fall rate, functional mobility, balance, health-related quality of life, and fear of falling (14). The authors suggest that an effective group-based exercise program consists of the following: (1) a similar group of individuals in terms of disease/impairment/age; (2) an easily accessible setting; (3) a physical therapist developed program with a supplemental HEP; and (4) a long-term or cyclic time frame to maintain benefits.

Patient adherence is one of the most important variables to determine the effectiveness of a group-based exercise program. Many factors impact adherence. Madureira et al. noted that patients are more likely to adhere when they belong to a social group with similar characteristics (18). Also, this social interaction seems to promote adherence to not only group-based exercise but to HEPs as well (18). Lord et al. further confirms this viewpoint and noted that group activities may facilitate long-term compliance to exercise programs, while also increasing enjoyment and social interaction (19).

Residents in a senior living community may share similar demographics and have the context to support social interactions. A senior living community is defined as a facility that provides nursing care, meals, and housekeeping. This type of setting may be ideal to achieve high adherence rates to a group-based exercise program. However, these residents do not all present with the same functional abilities. To significantly improve balance and decrease risk of falls, balance must be challenged from a moderate to high extent (12). This can pose a challenge when designing a “one size fits all” group-based exercise class aimed at reducing falls because the exercises may prove too challenging or not challenging enough for all of its participants. In a typical senior living community, group-based exercise classes are offered sparingly, instructed by untrained/unqualified staff members, and classes are designed so that all residents can participate, regardless of functional abilities. This type of setting creates a clear need for a program designed by a physical therapist that is evidence-based, instructed by a health-care professional, and is able to provide appropriate dosing and challenge to its participants.

In an effort to promote wellness and maintain an optimal level of function in the older adult population, we have implemented a program in a senior living community that provides therapy services when medically necessary as well as group-based exercise classes twice per week. Prior to the project, two different classes were offered. There were no objective measures to identify fall risk levels and participants were subjectively placed into one of the classes by an exercise physiologist. The purpose of the classes was to improve strength, mobility, and balance. Exercises in the classes were chosen at the discretion of an exercise physiologist.

Given the significant challenges of managing fall risk past a physical therapy episode of care, and the compelling evidence for the efficacy of group-based exercise classes to address risk, the current exercise classes were identified as an opportunity to expand fall risk reduction services. If the classes could integrate evidence-based fall prevention exercises, then these classes could be the foundation of a fall reduction program that is feasible, evidence-based, and provides maximum value to its participants and the facility.

The purpose of this study was to translate research findings into a “real world” setting, and demonstrate the efficacy of integrating evidence-based fall prevention exercises into pre-existing exercise classes at a senior living facility as a “proof of concept” model for future programing.

## Materials and Methods

**Figure 1:**
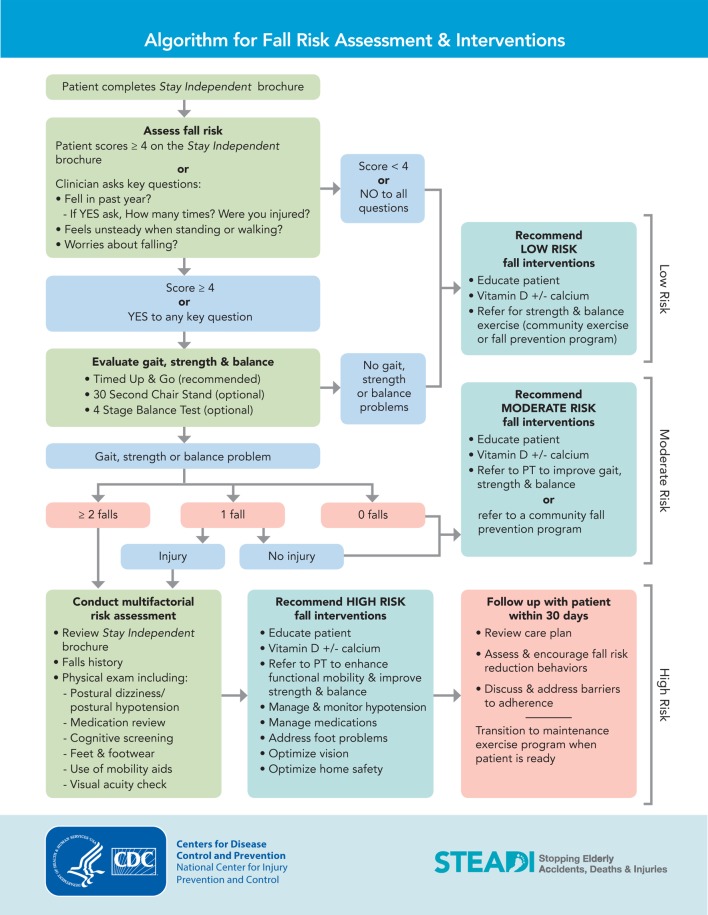
**STEADI algorithm for fall risk assessment and intervention**. Available from: https://www.cdc.gov/steadi/pdf/algorithm_2015-04-a.pdf.

A priority in this project was to appropriately stratify participants into a low, medium, or high-intensity group-based exercise class to insure the appropriate intensity of exercises was provided to participants. The classification schema that was implemented is one that was developed by the CDC, entitled “Algorithm for Fall Risk Assessment and Intervention.” The algorithm is part of a program developed by the CDC entitled “Stopping Elderly Accidents, Deaths, and Injuries,” (STEADI) (20) (Figure 1).

To achieve the above, the following were completed. First, in order to improve the quality of the group-based exercise classes, the current classes were evaluated to identify offerings that were not evidence-based. After evaluation, the classes were updated to integrate evidence-based exercises. Functional outcome measures were implemented to evaluate the program’s effectiveness, determine fall risk, and establish cutoffs as supported in the literature and the STEADI algorithm. Prior to beginning the class, participants were appropriately stratified into one of three classes based on the results of their functional outcome measures and fall history. Data were periodically collected to analyze functional outcomes, as well as number of falls and hospitalizations. These data were then compared to a similar site.

### Participants

Residents from a senior living community as well as community-dwelling older adults were included in the project. Inclusion criteria required participants to be age 65 years or older and to be ambulating without assistance. Full-time independent ambulators were chosen to ensure high challenge balance exercises could be implemented safely in a group setting. Participants were excluded from the project if they had a diagnosis of dementia. A total of 24 people met the inclusion criteria and consented to participation. There were 16 senior living community residents and 8 community dwellers. There were 9 males and 15 females. All participants were attending group-based exercise classes at the senior living community prior to the project. This project was an internal quality improvement project to determine the efficacy of the current exercise programs. All participants volunteered for the exercise classes. As part of the screening process, participants sign a consent form to be in the class and to have outcomes data collected for research purposes.

### Procedure

The components of the current exercise programs were assessed. Prior to the project, there were two group-based exercise classes being offered at a senior living community. Participants included residents of the facility and community-dwelling older adults who attended the class. Each class was 1 h in duration, and classes were offered twice a week. The two classes differed in the level of intensity. There was a “low intensity” and “high intensity” class.

The exercises performed in the low intensity class consisted of a combination of upper and lower extremity exercises performed in a seated position. Functionally, individuals in the low intensity class were a mix of people who ambulated with assistance and non-ambulators in wheelchairs.

Given the functional abilities of all participants, the class was offered at an appropriate intensity to improve ROM and strength, while maintaining safety of all participants. No changes were made to the low intensity class. Therefore, data were not tracked from its participants.

The high-intensity class was assessed for evidence-based components. The high-intensity class consisted of a combination of seated and standing exercises (Table 1). It was determined that the current class did not provide the appropriate intensity of exercises to all of its participants because balance exercises were underutilized, participants were under challenged, the exercises maximize Sherrington’s factors, and the exercises were not evidence-based.

**Table 1:** **Exercises performed in the old and new group-based exercise classes**.

	Old high intensity	New high intensity	New medium intensity
Seated exercises	Long arc quads, straight leg lifts, overhead reaching, bilateral shoulder flexion	Cervical rotation, cervical retraction, ankle rotations	Cervical rotation, cervical retraction, ankle rotations
Standing exercises	Marching, sit to stand, hip abduction, hip extension, knee flexion, heel/toe raises	Mini squats, sit to stand, hip abduction, hip extension, marching, knee flexion, heel/toe raises	Mini squats, sit to stand, hip abduction, hip extension, marching, knee flexion, heel/toe raises
Balance exercises	Side-stepping, single leg stand	^a^Heel walking, toe walking, semi-tandem stance, tandem stance, single leg stance, sidestep walking, backward walking, tandem walking, high knees walking, sidestep with UE movement, backstep with UE movement	^b^Toe marches, heel marches, semi-tandem stance, tandem stance, single leg stance, slow marching in place, step forward/lateral/posterior, reaching forward, and overhead with narrow base of support

*^a^UE, upper extremity. Participants instructed to use UE support only as necessary*.

*^b^Participants instructed to use at least one UE support at all times*.

To best tailor the intensity of the class to the participant’s abilities, the high-intensity class was split into two separate classes, medium and high intensity. This approach allowed more specific balance exercises of varying intensities to be implemented that would appropriately challenge participants.

Before beginning the group-based exercise class, a physical therapist evaluated each patient on gait, strength, balance, and fall history. The evaluations were completed in private sessions using the 10-Meter Walk Test (gait speed), 30-Second Sit to Stand, Timed Up and Go (TUG), and fall history in the past year.

The 30-Second Sit to Stand measures functional lower extremity muscle strength (21). Normative values as well as cutoff scores for fitness standards to maintain physical independence have been published (21). According to the STEADI algorithm, any score below age norms indicates a risk of falling (20).

The TUG assesses mobility, balance, walking ability, and fall risk in older adults (22). Normative data are available for many commonly seen diagnoses and cut off scores indicate risk of falling (22).

The 10-Meter Walk Test, or gait speed, has shown to be predictive of dependence with ADL’s and IADL’s, predict the likelihood of hospitalization, assess the need for interventions to reduce falls risk, predict discharge setting after hospitalization, and classify community vs. homebound ambulators (23).

The participants’ fall risks were stratified into three categories: low, medium, and high. The stratification was completed using an algorithm similar to the one created by the CDC as a component of their STEADI tool kit (20).

Cutoff scores were used to stratify participants into three balance classes – high intensity, medium intensity, and low intensity (Table 2). Participants with a TUG score less than 12 s, gait speed greater than 0.8 m/s, and a history of 0 falls in the past year were stratified into the high-intensity class. Participants with a TUG score between 12 and 20 s, gait speed between 0.6 and 0.8 m/s, and a history of 0 or 1 fall without an injury in the past year were stratified into the medium intensity class.

**Table 2:** **Stratification criteria for group-based exercise class determined by gait speed, timed up and go, and falls in the past year**.

Exercise class	Gait speed (m/s)	Timed Up and Go (s)	Falls in past year?
High	>0.8	<12 s	0
Medium	0.6–0.8	12–20 s	0 or 1 without injury

Both classes shared the following components; 5 min seated warmup, 20 min of standing lower extremity strengthening, 5 min water break, and 30 min of balance exercises. The warmup, strengthening, and balance exercises were updated to incorporate exercises from the OTAGO Exercise Program. The OTAGO Exercise Program was chosen because it is an evidence-based program that is endorsed by the CDC and is proven effective in reducing falls by up to 35% when compared to a non-exercise control (24). It includes a strengthening section and a balance re-training section that match the structure of the current group-based exercise class. Table 3 summarizes the changes to the components of the high and medium intensity class, as well as the change in duration to each component.

**Table 3:** **Changes made to the time spent on each component in the high and medium intensity classes**.

Components	Before	After	
	
	High intensity (min)	Medium intensity (min)	High intensity (min)
Warm up	10	5	5
Sitting strengthening	20	None	None
Standing strengthening	20	20	20
Water break	5	5	5
Balance training	10	30	30

The difference between the medium- and high-intensity classes was the difficulty of balance exercises performed. Balance exercises were chosen that proved to be challenging and appropriate to the class. Participants were encouraged to progress the difficulty of the exercises when deemed safe and appropriate by the instructor. This was accomplished by reducing the amount of upper extremity support, closing eyes during static activities, or adding dynamic extremity movements.

In the high-intensity class, balance exercises included sensory integration training without upper extremity support, multidirectional stepping with dual tasking, and dynamic high-intensity balance exercises from the Otago Exercise Program. Participants were instructed to use upper extremity support on an as-needed basis (Table 1).

In the medium intensity class, balance exercises included sensory integration training with upper extremity support, multidirectional stepping with upper extremity support, static reaching outside of base of support, and dynamic balance exercises with upper extremity support as needed that were adapted from the OTAGO Exercise Program. Participants were instructed to use at least one upper extremity support for balance at all times to ensure safety (Table 1).

A physical therapist developed the curriculum for each of the classes. For the first 12 weeks of the project, the physical therapist worked with the exercise physiologist in instructing the classes. Once the physical therapist felt comfortable that the exercise physiologist could instruct the core components of the class with fidelity, the exercise physiologist began instructing the classes full time, and the physical therapist checked in periodically. A checklist was used at each class to ensure all of the exercises were performed. By the end of the project, the exercise physiologist was able to instruct all three classes.

As a control, data was compared to a control group in a different senior living community that offered classes with similar exercises aimed at improving strength and mobility. The classes were taught by an exercise physiologist and were of the same duration and frequency. There was one class that was offered two times per week. Participants were residents of the senior living community, and no community-dwelling older adults attended the class. Data were collected on the same outcomes and over the same time period as the intervention group. Class attendance was not tracked. The data were analyzed and compared to the intervention group to determine the effectiveness of the stratification and changes to the group-based exercise classes.

### Data Analysis

Follow up assessments of each participant were completed at 12 weeks and at 25 weeks. Number of falls, hospitalizations, physical therapy episodes of care, and attendance were tracked throughout the project. There was a 75% attendance requirement to be included in the data analysis. An attendance requirement was used to ensure participants were receiving close to the dosage required for a change in balance as supported in the literature.

To ensure the control and intervention group were similar at baseline, chi-square and *T*-tests were performed to compare sex, age, amount of community participants, and baseline functional outcome scores (TUG, gait speed, 30-Second Sit to Stand).

*T*-tests were performed to determine the mean change in the TUG, gait speed, and 30-Second Sit to Stand and to determine the significance between the intervention and control groups. Chi square tests were performed to determine the statistical significance of differences between the intervention and control groups.

## Results

**Figure 2:**
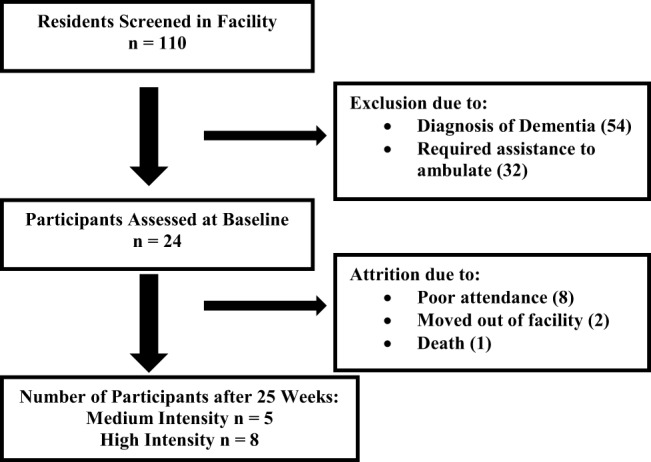
**Flow diagram of participants assessed at baseline, attrition, and 25-week reassessment**.

There were 110 residents living in the senior living community who were screened. Approximately half of the residents were already participating in the current group-based exercise classes. Twenty-four participants met the inclusion criteria of the medium (TUG 12–20 s, gait speed 0.6–0.8 m/s) and high-intensity (TUG < 12 s, gait speed > 0.8 m/s) class after being assessed at baseline (Figure 2). In order to be stratified into the medium or high-intensity class, each participant had to meet the criteria of both functional outcome measures. The mean age of all participants in the intervention group was 84.8 years (SD 5.2, 76–92). Of the 24 participants initially assessed, 11 were stratified into the high-intensity class and 13 were stratified into the medium intensity class. After the 25-week reassessment, 13 participants met the attendance requirement (75% of classes) for data analysis (Figure 2). There was an attrition of 11 participants. Eight participants did not meet the necessary attendance requirement. Reasons for poor attendance included illness (4), lack of motivation (2), and scheduling conflict (2). Two participants moved out of the facility. There was one death during the project that was unrelated to the exercise class. There were 17 participants assessed in the control building. The intervention group had five community members and the control group had none.

Initial testing was performed to compare demographics and baseline scores on functional outcome measures between the control and intervention groups (Table 4). Due to the small sample size for the project, the data from the medium and high-intensity participants were combined and then compared against the control. The mean age of the intervention group was less than the control group (*t*(14) = 2.56, *p* = 0.016). The intervention group had a higher proportion of males [30.8% (4/13)] vs. the control [11.8% (2/17)]. The intervention group consisted of five community-dwelling older adults. The intervention group performed significantly better on the TUG (*p* = 0.019) and 30-Second Sit to Stand *p* = 0.011, while there was only a slight difference in gait speed (*p* = 0.077). The high-intensity group scored most favorably on all three outcome measures.

**Table 4:** **Baseline demographics and functional outcome measures of control, medium intensity, and high intensity**.

Variable	Control	Intervention – medium intensity	Intervention – high intensity	Significance, *T*-values, chi square values
Age (SD)	89.9 (5.6)	86.8 (3.5)	83.5 (5.7)	*p* = 0.016, *t* = 2.56
Sex – female	15	8	8	
Sex – male	2	5	3	
Community participants	0	0	5	
Timed Up and Go	21.4	17.9	9.4	*p* = 0.019, *t* = 2.17
Gait speed	0.62	0.65	0.99	*p* = 0.077, *t* = 2.54
30-Second Sit to Stand	7.8	9.4	11.1	*p* = 0.011, *t* = 3.11

*p = p-value significant if <0.05*.

*Data from medium and high intensity combined for comparison to control group*.

Table 5 illustrates the changes in functional outcome measures in each group at the 12- and 25-week reassessment. Data from the medium- and high-intensity classes were combined for the analysis due to the small sample size in the project. The intervention group significantly improved in the TUG (*t*(12) = 3.73, *p* = 0.034), gait speed (*t*(12) = 5.96, *p* < 0.0001), and 30-Second Sit to Stand (*t*(12) = 4.06, *p* = 0.002). Outcomes improved at the 12-week reassessment and continued to improve until the final 25-week reassessment. The control group experienced a significant change in gait speed (*t*(14) = 2.85, *p* = 0.013) and the 30-Second Sit to Stand (*t*(14) = 2.63, *p* = 0.02), but not in the TUG (*t*(14) = 1.54, *p* = 0.146). There was no difference in change between the two groups for all three outcome measures. After the 25-week reassessment, two participants in the intervention group were re-stratified from the medium to the high-intensity class due to the improvements in their functional outcome measures.

**Table 5:** **Mean, significance, and between group significance of functional outcome measures at baseline, 12 weeks, and 25 weeks**.

Functional outcome measure	Control				Intervention				
	Mean at baseline	Mean at 12 weeks	Mean at 25 weeks	Significance of change (baseline vs. 25 weeks)	Mean at baseline	Mean at 12 weeks	Mean at 25 weeks	Significance of change (baseline vs. 25 weeks)	Difference in change between control and intervention from baseline to 25 weeks
Timed Up and Go	21.4	21.0	18.4	*p* = 0.146	13.5	11.2	10.4	*p* = 0.034	0.017, *p* = 0.99
Gait speed	0.62	0.65	0.77	*p* = 0.013	0.81	0.93	0.98	*p* < 0.0001	0.02, *p* = 0.73
30-Second Sit to Stand	7.8	8.8	9.5	*p* = 0.020	10.5	12.1	13.4	*p* = 0.002	1.2, *p* = 0.23

*p = p-value significant if <0.05*.

None of the participants in the intervention group experienced a fall during the study. The control group had 8 of the 17 participants fall, of which 3 participants fell multiple times bringing the total number of falls up to 16. Two individuals who fell required hospitalizations due to fractured wrists. None of the participants in the intervention group were hospitalized during the study. The control group had six of its participants hospitalized. One participant was hospitalized four times, making the total number of hospitalizations nine. Two participants in the intervention group required a physical therapy episode of care during the project. Eight participants required physical therapy in the control group.

## Discussion

The findings in this project suggest that participation in a 25-week group-based exercise class has a positive effect on strength, mobility, balance, gait speed, and fall risk.

Participants demonstrated significant improvements in the TUG, 30-Second Sit to Stand, and gait speed. The TUG is a comprehensive test that assesses mobility, balance, walking ability, and fall risk. At 25 weeks, the mean (10.4 s) exceeded the 12 s cutoff indicating a reduced risk for falling (25). The mean is also now within published age norms (26). The 30-Second Sit to Stand mean (13.4) is now above published age norms (11.9), further indicating reduced fall risk (27). The gait speed mean of 0.98 m/s was just below the 1.0 m/s cutoff associated with increased risk of falls (28). Mean scores improved from baseline by 0.17 m/s, which exceeds Perara et al.’s published minimal clinical important change (MCID) of 0.13 m/s, indicating a substantial meaningful change (29).

Participants in the intervention group did not experience a fall or hospitalization during the project. This result speaks to the importance of properly matching participants’ functional ability to the difficulty of the class. The control group had three participants fall multiple times. These participants may be frailer, in a downward functional spiral, and require immediate attention through physical therapy intervention and a lower intensity class to ensure their safety in a group setting.

Less physical therapy intervention was required in the intervention group. This may have been due to the higher functional wellness of the intervention group, which resulted in less need for physical therapy intervention. On the contrary, the control group may have required more physical therapy episodes of care due to the higher amount of hospitalizations and falls.

Baseline comparisons between the control and intervention group revealed significant differences. The current wellness model had been in place for 4 years in the intervention group and only 1 year in the control group. The higher dose of exercise received by the intervention group may explain why they scored higher on baseline testing. Dissection of the intervention group revealed that participants in the medium intensity class were scored similar to the control group on their functional outcome measures. The high-intensity class, which contained the five community-dwelling adults, scored the best on initial functional outcome measure testing.

There was no significant difference in the change in functional outcome measures between the control and intervention groups. At 12 weeks, the intervention group was demonstrating a more positive trend of improvement in all outcomes. By 25 weeks, both groups improved by similar amounts. A closer examination of the data revealed a few outliers in the control group which drastically improved over the course of the project which may have skewed the data, especially considering the low number of participants. It is also possible that most of the gains seen in the control group were due to the fact that 8 of the 17 of the participants received physical therapy during the project. The additional dosage of individualized exercise may have led to more improvements than if they were only attending the group-based exercise class.

The current wellness program has the exercise physiologist in each building choose which exercises to perform in their classes. It is possible that the current offerings in the control building are of an appropriate dosage and intensity to its participants. However, it is unknown what is being performed in other buildings with this program implemented. In order to ensure appropriate dosage in all buildings, the work completed in this project can now act as standardization for other programs to improve outcomes.

This project has implemented an algorithm for stratifying fall risk, implemented evidence-based group exercise classes, and improved outcomes through properly dosed exercises. Rather than subjectively being placed into classes, participants are now objectively stratified into an appropriate class based on their functional outcome measures. As a result, participants are receiving an intensity of balance exercises that is matched to their ability and which has been shown to maximally reduce falls. The group-based exercise classes now act as long-term supplements to the standard physical therapy plan of care, allowing clients to achieve the proper dosage of balance interventions as supported by the literature.

### Limitations

The main limitation in the project was the small sample size, which ultimately limited the statistical power of the results. The inclusion criteria cut the sample size down from 110 participants to 24 participants, and there was an attrition of 11 participants throughout the project. The attrition was mostly due to the high attendance requirement for analysis.

A second limitation was the length of the project. Participants came very close to matching the dose recommendations proposed by Sherrington. When each hour long class was dissected, it included a 5-min warmup and 5-min water break. Therefore, the classes consisted of 50 min of true balance training, and participants were falling just short of the suggested 2 h per week. The final reassessment was performed at 25 weeks because it matched the required time to reach the 50 h of balance training proposed by Sherrington (12). However, only two participants attended 100% of the classes. In the future, the class may need to be lengthened if kept at twice per week or increased to three times per week.

The literature does support the use of a supplemental HEP. In an effort to reduce the burden placed on the participants, a HEP was not administered. In the future, it may be valuable to administer a HEP at the start of the program.

## Conclusion

As the health-care system and reimbursement system continues to evolve, so must physical therapists to ensure that clients continue to have access to care of the highest value. By transitioning to a wellness model of health care, a shift in mindset occurs that places fall prevention to the forefront of the discussion when it comes to improving outcomes and reducing falls, hospitalizations, and costs.

This project acts as a proof of concept. The project’s framework can be used to model programs in similar settings and institutions looking to reduce falls. The project has synthesized many aspects of the literature to develop a deliverable product that is evidence-based on many levels including the screening and stratification of fall risk and proper dosage and intensity of exercises.

## Author Contributions

AH, TS, and WD – concept and design, analysis and interpretation of data, and manuscript preparation. AC – analysis and interpretation of data.

## Conflict of Interest Statement

This research was conducted in the absence of any commercial or financial relationships that could be construed as a potential conflict of interest.
